# Factors associated with noninvasive ventilation usage in patients with hypoventilation disorders

**DOI:** 10.1093/sleepadvances/zpae046

**Published:** 2024-07-11

**Authors:** Riley Forbes, Brett Duce, Craig Hukins, Claire Ellender

**Affiliations:** Faculty of Medicine, University of Queensland, Brisbane, Australia; Department of Respiratory & Sleep Medicine, Princess Alexandra Hospital, Brisbane, Australia; Department of Respiratory & Sleep Medicine, Princess Alexandra Hospital, Brisbane, Australia; Institute of Health and Biomedical Innovation, Queensland University of Technology, Brisbane, Australia; Faculty of Medicine, University of Queensland, Brisbane, Australia; Department of Respiratory & Sleep Medicine, Princess Alexandra Hospital, Brisbane, Australia; Faculty of Medicine, University of Queensland, Brisbane, Australia; Department of Respiratory & Sleep Medicine, Princess Alexandra Hospital, Brisbane, Australia

**Keywords:** noninvasive ventilation, neuromuscular disease, adherence, adherence, usage

## Abstract

**Study Objectives:**

The objective of this study was to investigate the association between demographic, clinical, and interface factors and noninvasive ventilation (NIV) usage.

**Methods:**

A retrospective cohort analysis of 478 patients prescribed NIV from 2013 to 2021 was performed. Demographic factors, clinical indications for NIV, and interface factors were collected, and linear regression was conducted to evaluate the association between these variables and NIV usage (hour/night).

**Results:**

The average usage of the cohort was 6.5 hour/night ± 4.6, with an average age of 57 years ± 16 and body mass index (BMI) of 40.5kg/m^2^ ± 14.7. The cohort was mostly male (*n* = 290, 60.6%). The most common indications for NIV prescription were high-pressure requirement for obstructive sleep apnea (HPR, *n* = 190, 39.7%), neuromuscular disease (NMD, *n* = 140, 29.3%), and obesity hypoventilation syndrome (OHS, *n* = 111, 23.2%). A diagnosis of NMD was a significant predictor of higher NIV usage (8.0 ± 6.1 hour/night) in multivariate analysis (*p *= .036). The HPR subcohort had the lowest usage of all indications. Age and BMI did not predict usage. A nasal interface (*p* < .01) and lower expiratory positive airway pressure (EPAP) setting (*p* < .001) were associated with increased NIV usage.

**Conclusions:**

This study highlights the multifaceted nature of NIV usage. Where demographic factors were not consistent predictors of usage, interface, and clinical indication were associated with usage. These findings highlight that the HPR users are a group at risk of low usage.

**Statement of Significance**

The efficacy of noninvasive ventilation (NIV) is strongly associated with usage, yet the factors influencing adherence are not fully understood. This study investigates the association between demographic, clinical, and interface factors with NIV usage, with a view to better understanding groups who would benefit from increased adherence support. A diagnosis of neuromuscular disease was a significant predictor of higher usage. Patients with a high-pressure requirement for obstructive sleep apnea or obesity hypoventilation syndrome were at higher risk of poor usage. Clinicians treating patients on NIV who are struggling with usage could consider changing to a nasal interface and titrating settings to the lowest safe and effective expiratory positive airway pressure as these factors predicted increased NIV usage.

Noninvasive ventilation (NIV) is commonly indicated to treat respiratory failure due to obstructive pathologies (13.2%), restrictive pathologies (9.5%), neuromuscular disease (30.2%), and obesity hypoventilation syndrome (OHS; 31%) [1]; while respiratory failure is a common feature, these diseases are very heterogeneous. In patients with obstructive sleep apnea whose breathing is inadequately controlled using maximum continuous positive airway pressure (CPAP) therapy, NIV is often indicated. NIV provides respiratory support with positive pressure through a sealed interface without the need for intubation, aiming to stabilize or improve respiratory insufficiency. NIV use is supported in chronically hypercapnic chronic obstructive pulmonary disease (COPD) patients [2], neuromuscular and chest wall disorders [3], as well as a variety of other indications [4]. NIV effectively reduces mortality in patients with COPD who are hypercapnic and experience frequent exacerbations with respiratory failure with reported hazard ratios from 0.24 to 0.49 [5, 6]. Furthermore, a 2020 meta-analysis of 51 085 patients with COPD demonstrated that domiciliary NIV is associated with a lower risk of mortality (OR = 0.66), all-cause hospital admission (OR = 0.22), and intubation (OR = 0.34) [7].

Long-term NIV usage has not been well defined in the literature, and the threshold of “adequate” versus “inadequate” usage varies between studies. It appears NIV usage of more than 5 hours per night is associated with reductions in hospitalizations and mortality [8–10]. Jeganathan et al. observed that a minimum threshold of 4 hour/night was required to demonstrate improvements in CO_2_ and daytime sleepiness [11]. In amyotrophic lateral sclerosis (also known as motor neuron disease), survival benefits and preservation of respiratory function are seen with usage greater than 4 hours per night [12, 13]. Factors influencing use of NIV are poorly understood compared to CPAP therapy. In a study of 1746 patients, NIV usage was reported to be 7.1 hours per night for neuromuscular disease (NMD, *n* = 397) and 5.8 hours per night for OHS (*n* = 515) [10]. Smaller-scale studies [11] support this usage trend.

There is inconsistent data surrounding predictors of NIV usage, for example, increased age was associated with improved usage in three studies [14–16], but a fourth study found no association [17]. Increased body mass index (BMI) has been consistently associated with lower usage [9, 14, 18]. Household income was found to be significantly associated with usage in one study of Duchenne’s muscular dystrophy [19], but in a second, broader study the relationship was not significant [19, 20]. In the study of Slot et al., they identified that patients living with a partner were almost five times more likely to adhere to treatment [17]. Mental health diagnoses appear to be associated with poor usage; for example, people with depression [17, 18] and schizophrenia have been shown to have lower NIV usage [16]. The type of mask has not been demonstrated to affect usage [15]. Interface and technical difficulties have been demonstrated to predict cessation [14]. Patient-reported reasons for cessation commonly include interface difficulties [21].

Given the conflicting findings, the question remains: which patient and treatment factors are associated with poor NIV usage? This information can directly inform clinical practice by directing clinicians and services to target supportive interventions for more vulnerable groups. This study aimed to assess the relationships between demographic, clinical, and interface data and NIV usage in a cohort of patients prescribed long-term domiciliary NIV.

## Methods

A retrospective, single-center, consecutive cohort study of patients on NIV seen at a tertiary sleep disorders center in Australia was performed following ethical approvals from Metro South Research Governance under HREC/2022/QMS/90051. Data were collected from a bespoke Filemaker Pro database where comprehensive clinical and equipment data are entered contemporaneously (Claris International Inc, Cupertino, California). Data collection were restricted from 2013 to 2021. Considering that the SARS-CoV-2 wave did not occur in this clinical environment until March 2022, this data represents prepandemic usage.

**Figure 1.**
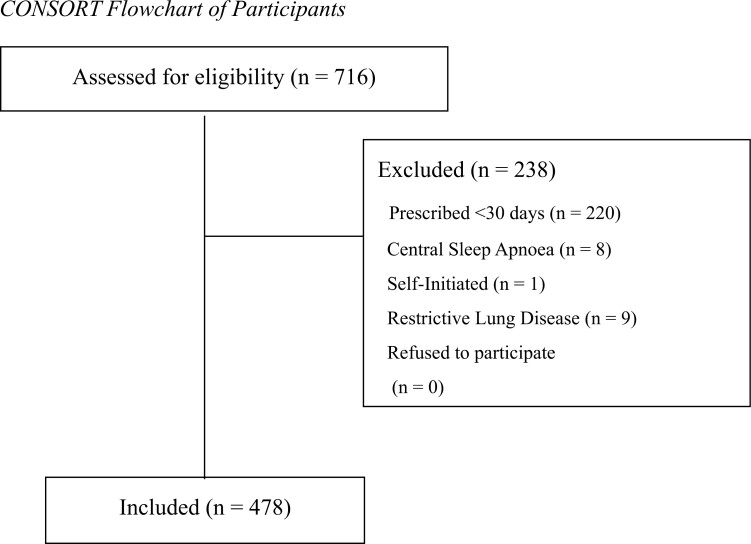
CONSORT flowchart indicating reason for exclusion among assessed participants.

The inclusion criteria were age greater than 18 years and history of NIV usage within the service. Both incident and prevalent patients were included. Exclusion criteria were acute inpatient NIV, a diagnosis of central sleep apnea, or prescription of NIV for less than 30 days, as shown in the CONSORT flowchart in Figure 1.

NIV usage was determined via manual hour meter recording or AirView^®^ (ResMed; San Diego, CA, USA, Version 4.43.0-24.0.0) download at the time of the last clinic appointment. Self-reported questionnaires were included, in this instance the Epworth Sleepiness Scale (ESS).

### Statistical analysis

The indication for NIV was categorized as (1) high-pressure requirement for OSA (HPR), (2) neuromuscular disease (NMD), (3) OHS, (4) obstructive lung disease (OLD) and, (5) restrictive lung disease. The HPR subcohort included patients with OSA that required greater than 20 cm H_2_O that have been prescribed NIV. The cohort of restrictive lung disease patients was not included in the final cohort due to the small numbers (9).

Normality was assessed with the Shapiro–Wilk test. Most variables were non-normally distributed, so data were compared using the Mann–Whitney U, Kruskal–Wallis, Dunn, and Chi-Square tests as appropriate. Where multiple comparisons were conducted, Bonferroni adjustments were applied. Linear regression was used to evaluate the relationships between continuous variables.

Socioeconomic status (SES) was defined using deciles derived from Australian Bureau of Statistics (ABS) data, namely the Index of Relative Socio-Economic Advantage and Disadvantage (IRSAD) [22]. Deciles between 1 and 10 are categorical, with a score of 10 indicating little disadvantage and a score of 1 indicating significant disadvantage. Time on therapy *was* a priori controlled for in a multivariate analysis. Statistical analyses were performed with IBM SPSS Statistics version 29, and the PROCESS macro version 4.2 was used for mediator/moderator analysis [23].

## Results

Four hundred and seventy-eight patients were included in the study, with an average age of 57 ± 16 years. Among these, 290 (60.6%) were male, and the remainder were female. The average IRSAD was 5.4 ± 2.8 decile, with 29 (6.1%) of the patients identifying as Aboriginal and/or Torres Strait Islander. The cohort was grouped into prespecified indications for NIV: 190 (39.7%) high-pressure requirements for OSA, 140 (29.3%) for neuromuscular disease, 111 (23.2%) for OHS, and 37 (7.7%) for OLD. The demographic and clinical characteristics of the patients are summarized in Table 1. The median time on NIV therapy was 20.6 (39.2) months.

**Table 1.** Demographic, Clinical, and Interface Data Segmented by Indication for Noninvasive Ventilation

	All	HPR	NMD	OHS	OLD	*P*-value
	(*n* = 478)	(*n* = 190)	(*n* = 140)	(*n* = 111)	(*n* = 37)	
Male, (%)	290 (60.6%)	120 (63.2%)	91 (65.0%)	49 (44.1%)	23 (62.2%)	<.001
Age, y	59.6 (21.1)	61.8 (20.7)	54.6 (24.2)	55.9 (17.1)	68.9 (10.9)	<.001
ATSI, (%)	29 (6.1%)	9 (4.7%)	7 (5.0%)	14 (12.6%)	2 (5.4%)	.122
IRSAD,	5.0 (5.0)	5.0 (6.0)	7.0 (4.8)	5.0 (5.0)	6.0 (6.0)	.019
BMI,	39.4 (19.5)	41.7 (14.9)	29.5 (13.8)	50.1 (19.4)	27.9 (15.3)	<.001
ESS,	9.0 (10.0)	11.0 (10.0)	8.0 (11.0)	10.0 (7.0)	7.0 (7.5)	<.001
Therapy time, mo	20.6 (39.2)	22.4 (48.4)	17.1 (34.3)	20.5 (29.9)	24.1 (37.1)	.596
Usage >6 h, (%)	267 (55.9%)	97 (51.1%)	92 (65.7%)	59 (53.2%)	21 (56.8%)	.045
Usage, h/n	6.6 (6.0)	6.0 (5.1)	8.1 (7.9)	6.2 (5.6)	7.0 (6.6)	.004
ST mode, (%)	177 (37.0%)	44 (23.2%)	80 (57.1%)	35 (31.5%)	15 (40.5%)	<.001
IPAP, (cmH2O)	22.0 (6.0)	22.0 (4.0)	17.8 (7.3)	24.0 (3.0)	18.0 (4.0)	<.001
EPAP, (cm H_2_O)	14.0 (8.0)	17.0 (5.6)	10.0 (7.0)	18.0 (5.0)	10.0 (5.2)	<.001
Backup rate, brpm	12 (2)	12 (0)	12 (2)	12 (2)	12 (0)	.784
Nasal interface, (%)	86 (18.0%)	36 (18.9%)	38 (27.1%)	12 (10.8%)	4 (10.8%)	.009

HPR, high-pressure requirement OSA; NMD, neuromuscular disease; OHS, obesity hypoventilation syndrome; OLD, obstructive lung disease; ATSI, Aboriginal and/or Torres Strait Islander status; IRSAD, Index of Relative Socio-Economic Advantage and Disadvantage; BMI, body mass index; ESS, Epworth Sleepiness Scale; ST mode, the proportion of patients requiring a backup rate; IPAP, inspiratory positive airway pressure; EPAP, expiratory positive airway pressure. *P* values indicate statistical significance across groups.

### Indications for NIV and group differences

There were significant sex differences between groups, with a higher proportion of females (*n* = 62, 65.9 %) in the OHS group (*p* < .001). Average age across groups was significantly different; the OLD group had a median age of 68.9 (10.9) years, which was significantly older than those in the high-pressure requirement for OSA (61.8 (20.7) years, *p* = .007), neuromuscular disease (54.6 (24.2) years, *p* < .001), and OHS (55.9 (17.1) years, *p* < .001) groups. The neuromuscular disease group had a lower BMI 29.5 kg/m^2^ (13.8) than the high-pressure OSA group (41.7 kg/m^2^ [14.9]) and OHS group (50.1 kg/m^2^ (19.4), *p* ≤ .001).

A significant association was found between NIV mode and its indication. More patients in the neuromuscular disease group were in spontaneous/timed mode (ST; *p* ≤ .001) and more in the high-pressure requirement group in spontaneous mode (S; *p* ≤ .001), as detailed in Table 1. Differences in NIV settings, including Expiratory Positive Airway Pressure (EPAP) and Inspiratory Positive Airway Pressure (IPAP), were significant between groups. Mask type also showed a significant correlation with the indication for NIV (*p* = .009).

ESS at the initiation of therapy was higher in the high-pressure requirement group compared to the neuromuscular disease (*p* = .014) and OLD (*p* = .01) groups.

### Predictors of usage

**Figure 2.**
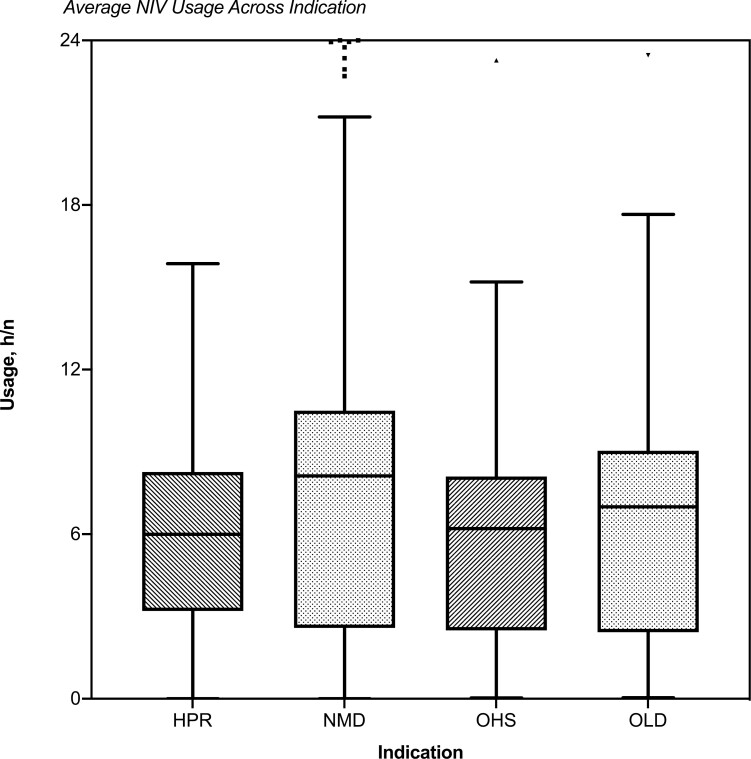
Tukey’s method to compare average nightly noninvasive ventilation usage across four patient groups; HPR; high-pressure requirement OSA, NMD, neuromuscular disease; OHS, obesity hypoventilation syndrome; OLD, obstructive lung disease.

The HPR group had a median usage of 6.0 hour/night and 51.1% > 6 hour/night, the NMD group 8.1 hour/night and 65.7% > 6 hour/night, the OHS group 6.2 hour/night and 53.2% > 6 hour/night, and the OLD group 7.0 hour/night and 56.8% > 6 hour/night. Single-variable regression analyses are described in Table 2 and in Figure 2. The indication for NIV had a significant association with NIV usage: R^2^ = 0.042, F (1, 476) = 6.84, *p* < .001. Patients from the neuromuscular group demonstrated a particularly strong association β = 2.16, (95% confidence interval [CI]: 1.16 to 3.16, *p* < .001).

**Table 2.** Single Variate Analysis for Predictors of Usage

Independent variable	*R* ^*2*^	*F*	*β*	95% CI		*P-*value
				LL	UL	
Indication	0.042	6.84				<.001
HPR	0.016	7.58	−1.19	−2.04	−0.341	.006
NMD	0.040	19.78	2.04	1.14	2.94	<.001
OHS	0.005	2.39	−0.779	−1.77	0.211	.123
OLD	0.000	0.000	0.015	−1.55	1.58	.985
IRSAD, deciles	0.04	0.77	−0.066	−0.081	0.213	.380
Age, y	0.01	4.94	−0.101	−0.56	−0.003	.027
BMI, kg/m^2^	0.026	12.85	−0.051	−0.023	−0.080	<.001
ESS	0.022	10.64	−0.108	−0.173	−0.043	.001
Therapy time, mo	0.016	7.51	0.018	0.005	0.032	.006
ST Mode	0.014	6.78	1.15	0.281	2.01	.010
IPAP (cm H_2_O)	0.016	7.50	−0.125	−0.215	−0.035	.006
EPAP (cm H_2_O)	0.033	16.21	−0.160	−0.238	−0.082	<.001
Nasal interface	0.012	6.72	1.42	0.34	2.49	<.010

HPR, high-pressure requirement for OSA; NMD, neuromuscular disease; OHS, obesity hypoventilation syndrome; OLD, obstructive lung disease; IRSAD, Index of Relative Socio-Economic Advantage and Disadvantage; BMI, body mass index; ESS, Epworth Sleepiness Scale; Time, duration of therapy; ST mode, the proportion of patients requiring a backup rate; IPAP, Inspiratory Positive Airway Pressure; EPAP, Expiratory Positive Airway Pressure. The table reports the coefficient of determination (R²), F-statistic (F), regression coefficient (β), 95% confidence interval (CI) for β (with lower limit [LL] and upper limit [UL]), and the *p*-value for each analysis.

Increasing age significantly predicted decreased NIV usage, β = −0.101, (95% CI: −0.56 to −0.003, *p* = .027,). Increasing BMI was significantly predictive of lower NIV usage, β = −0.051, (95% CI: −0.023 to −0.080, *p* < .001). Neither sex nor Aboriginal and/or Torres Strait Islander status were significantly associated with NIV usage. IRSAD did not demonstrate any significant relationship with usage β = −0.066, (95% CI: −0.081 to 0.213, *p* = .38). Increasing ESS at therapy initiation predicted lower usage β = −0.108, (95% CI = 0.043 to 0.173, *p* = .001). Increasing time on therapy significantly predicted usage β = 0.018, (95% CI: 0.005 to 0.032, *p* = .006). Using ST mode rather than S predicted increased usage β = 1.15, (95% CI: 0.281 to 2.01, *p* = .010). Increasing IPAP predicted lower usage β = −0.125, (95% CI: −0.215 to −0.035, *p* = .006). Similarly, increasing EPAP predicted lower usage β = −0.160, (95% CI: −0.238 to −0.082, *p* < .001). Nasal mask usage predicted increased usage β = 1.42, (95% CI: 0.34 – 2.49, *p* = .01).

### Multivariate analysis

Adjusting for age, BMI, ESS, therapy time, ST mode, IPAP, EPAP, and use of nasal interface, relationship between neuromuscular disease and usage was significant. Compared to high-pressure requirement OSA, belonging to the neuromuscular group was associated with an increase of 1.26 hours of usage per night (β = 1.26, *p* = .036). A one-unit increase in ESS was associated with a decrease of 4.68 minutes of usage per night (β = −0.078, *p* = .021). A 1 cmH2O increase in EPAP was associated with an 11-minute decrease in usage per night (β = −0.184 *p* = .045). Multivariate analysis is described in Table 3.

**Table 3.** Multivariate Analysis for Predictors of Usage

Independent variable	Slope	Standard error	*t* ratio	*P*-value
*Indication*				
HPR	—	—	—	—
NMD	1.26	0.600	2.10	.036
OHS	0.328	0.558	0.588	.557
OLD	−0.696	0.878	1.26	.207
Age, y	−0.018	0.014	−1.30	.195
BMI	−0.032	0.017	−1.89	.059
ESS	−0.078	0.034	−2.322	.021
Therapy time	0.020	0.007	3.01	.003
ST mode	−0.039	0.488	−.080	.937
IPAP	0.163	0.488	1.66	.098
EPAP	−0.184	0.092	−2.009	.045
Nasal interface	0.677	0.536	1.26	.207
Constant	7.74	1.71		
R^2^	0.107			
F ratio	5.02			
*P*-value	<0.001			
n	478			

HPR, high-pressure requirement for OSA; NMD, neuromuscular disease; OHS, obesity hypoventilation syndrome; OLD, obstructive lung disease; IRSAD, index of relative socioeconomic advantage and disadvantage; BMI, body mass index; ESS, Epworth Sleepiness Scale; Therapy time, duration of therapy, ST mode, the proportion of patients requiring a backup rate; IPAP, inspiratory positive airway pressure; EPAP,; expiratory positive airway pressure.

### Mediators and moderators of NIV usage

It was found that IPAP pressure had a significant indirect effect on usage through mask type β = 0.15 (95% CI = 0.002 to 0.39, *p* = .014), suggesting that pressure plays a part in the relationship between mask type and usage. Neuromuscular patients in ST mode demonstrate a significant increase in usage β = 3.23 (95% CI: 1.87 to 4.59, *p* < .001).

NIV usage in the high-pressure requirement for OSA cohort is moderated by socioeconomic status as measured by IRSAD, β = 0.31 (95% CI = 0.015 to 0.61, *p* < .001). Relative socioeconomic advantage normalizes the usage in the HPR subcohort.

## Discussion

This study aimed to assess the relationships between demographic, clinical, and interface factors and NIV usage in a cohort of long-term users. The cohort included 478 individuals and encompassed 31 months of NIV therapy, representing a large and diverse population of long-term users. The average usage of NIV in this cohort was 6.5 hour/night which is consistent with broader literature [10, 11]. The cohort represents a broad spectrum of socioeconomic status, with a median IRSAD of 5.0 (5.0) decile.

There were significant differences in demographics in subcohorts. The NMD group was distinctive compared with other indications, tending to have a lower BMI, being younger, and having a non-sleepy phenotype. The HPR and OHS groups had similar demographic factors. Both the HPR and OHS subcohorts demonstrated a higher BMI. Both subcohorts were phenotypically sleepy and utilized higher pressures. The OLD sub-cohort demonstrates an older age than other groups. Usage of NIV was significantly different between groups. Where NMD patients utilized NIV more than other groups, usage in the HPR sub-cohort was significantly lower. The NMD sub-cohort was more likely to use ST-mode and nasal interfaces. Moderator analysis demonstrated that ST mode drives increased usage in the NMD sub-cohort. Considering that the necessity for ST mode may reflect greater disease severity, this result suggests disease severity might drive increased usage. Usage in the HPR sub-cohort was lower, though this result was not significant in multivariate analysis.

Age initially emerged as a significant positive predictor of usage but was not significant in multivariate analysis, reflecting the complicated relationship between age and usage found in the literature [14, 15, 17]. In direct analysis, the IRSAD as a measure of socioeconomic status did not significantly correlate with NIV usage. Although this could reflect a shortcoming of the IRSAD to adequately capture or emphasize the pertinent socioeconomic factors that determine usage, perhaps this reflects a strength of the healthcare system in addressing equity. In moderation analysis, it was demonstrated in the HPR sub-cohort that poor usage was minimized by relative socioeconomic advantage. This would suggest that in HPR patients with socioeconomic advantage there is no negative effect on usage. The mechanism of this is unclear. The fact that this moderator relationship was isolated to the HPR sub-cohort is of uncertain significance. It could be speculated that in other subcohorts the ancillary benefits available address components of socioeconomic status that impact usage, for example, carer support at home. Similarly, socioeconomic factors appear to play a significant role in usage of patients with OSA on CPAP [24]. A higher EPAP was significantly associated with poorer usage in multivariate analysis. This might reflect a common observation that higher-pressure settings are uncomfortable. Alternatively, this may simply reflect a correlative relationship. The effect of mask type on usage is significant when controlling for indication for NIV (*p* = .034), though it is no longer significant in complete multivariate analysis. Interestingly, we identified that the effect of mask type on NIV usage was partially mediated by IPAP. This suggests that a lower pressure might explain the effect of mask type on NIV usage. There are multiple plausible explanations for this finding. It could be speculated that usage of nasal masks results in a lower pressure requirement similar to pressure requirements in CPAP [25]. Practical considerations often influence interface selection. For example, many patients are initiated on NIV in the Emergency Department where oronasal interfaces are often used by default. Alternatively, perhaps there are yet unidentified factors that would predict each of a lower pressure requirement, nasal mask usage, and lower usage. This analysis was not able to utilize leak or comfort parameters due to limited reporting. Such factors could potentially interrogate the role of comfort and interface performance in determining usage. Although this analysis supports the use of nasal masks, interface type is largely determined by individual factors such as patient preference, comfort, and mask fit. Increasing subjective daytime sleepiness as measured by the ESS was predictive of lower usage in multivariate analysis (*p* = .021). Notably, the ESS is a nonspecific scale that is unable to differentiate between causes for a higher score. This might reflect a direct causal relationship between subjective daytime sleepiness and usage, but could potentially reflect a common cause relationship, that subjective daytime sleepiness and lower usage are predicted by the same independent variable. Regardless, there is significant utility of subjective daytime sleepiness as a risk stratification tool. Our analysis demonstrates that the individuals at greatest risk of poor usage are those who are highly symptomatic (as measured by the ESS) people who require both high EPAP and full-face masks. This research serves to inform equipment programs and titration protocols. Although retrospective, it suggests that there could be a limited clinical benefit to dropping pressure settings for comfort.

The strengths of this study included the wide range of patients across various demographics and clinical backgrounds and adequate sample size in each indication group. A range of clinically relevant factors were assessed. The cohort had an average follow-up on therapy of 31 months; thus, a strength was our long-term usage data. With a retrospective design, there are several limitations, and the cohort was treated at a single site. Exploration of potentially critical factors, including psychological determinants of usage, the presence of carer or family support, patient opinions, or comfort data, was not included. Exploration of these factors could provide insight into further patient-centered and modifiable determinants of usage. This investigation considers patient usage as consistent despite variability. Further research might consider usage as fluctuant and aim to identify factors that cause changes in usage.

The implication of this study is that not all NIV users are the same. The next step will be to maintain current programs in place to continue to support neuromuscular patients’ use of NIV; however, it appears that some customized support is needed to improve usage in high-pressure requirement patients with OSA. Where neuromuscular patients are struggling with NIV, it may be useful to trial a nasal mask if a full-face mask has previously been used.

## Conclusions

This study highlights the complexity of factors influencing usage of NIV. Demographic factors like age and socioeconomic status showed no consistent impact on usage. Mask type and NIV settings emerged as important determinants of usage. Patients requiring NIV for high-pressure requirement OSA may be vulnerable to poor NIV usage, especially at high expiratory pressures and may warrant additional support to improve usage.
